# Invasive Tracheobronchial Aspergillosis: A Fatal Complication in a Patient With Treated Mediastinal Lung Adenocarcinoma

**DOI:** 10.7759/cureus.70024

**Published:** 2024-09-23

**Authors:** Takato Ikeda, Toyoshi Yanagihara, Maiya Chen, Rei Sanai, Yusuke Osaki, Momoka Kikushima, Mikiko Aoki, Yoshihiro Hamada, Makoto Hamasaki, Naoki Hamada, Masaki Fujita

**Affiliations:** 1 Department of Respiratory Medicine, Fukuoka University Hospital, Fukuoka, JPN; 2 Department of Pathology, Fukuoka University School of Medicine, Fukuoka, JPN; 3 Pathology, Faculty of Medicine, Fukuoka University, Fukuoka, JPN; 4 Department of Respiratory Medicine, Fukuoka University School of Medicine, Fukuoka, JPN

**Keywords:** aspergillus, aspergillus tracheobronchitis, bronchitis, invasive pulmonary aspergillosis, respiratory tract infections

## Abstract

Invasive tracheobronchial aspergillosis (ITBA) is a rare but severe form of invasive aspergillosis. This report presents a fatal case of ITBA in a 75-year-old man with a complex medical history including mediastinal lung adenocarcinoma, radiation pneumonitis, and pulmonary nocardiosis. The patient was admitted with worsening dyspnea and chest imaging revealed severe airway stenosis. Initially suspected to be cancer recurrence, post-mortem examination confirmed ITBA caused by *Aspergillus penicillioides*. Histopathological findings showed fungal invasion of the tracheobronchial tree with destruction, obstruction, and perforation of the airways. Multiple risk factors likely contributed to the development of ITBA in this patient, including diabetes, chronic obstructive pulmonary disease (COPD), long-term steroid use, prior COVID-19 infection, and a history of radiation therapy. This case highlights the diagnostic challenges of ITBA, particularly in patients with multiple comorbidities and a history of malignancy. It emphasizes the importance of considering fungal infections in the differential diagnosis of airway obstruction in high-risk patients.

## Introduction

Aspergillus species are ubiquitous environmental fungi capable of causing a diverse spectrum of respiratory diseases. These conditions range from relatively benign to life-threatening invasive infections, with their manifestation largely dependent on the complex interplay between the fungus and the host's immune system [1].

Respiratory aspergillosis can be broadly categorized into three main types: allergic pulmonary diseases, saprophytic infections, and invasive diseases [2]. Allergic manifestations, such as allergic bronchopulmonary aspergillosis and hypersensitivity pneumonitis, arise from hypersensitivity reactions to Aspergillus antigens, typically in individuals with atopic predispositions. Saprophytic infections, including aspergilloma and endobronchial aspergilloma [3], occur when Aspergillus colonizes pre-existing lung cavities or airways without tissue invasion, often in patients with structural lung abnormalities.

Invasive aspergillosis represents the most severe form of the disease spectrum. It encompasses invasive pulmonary aspergillosis (IPA) and invasive tracheobronchial aspergillosis (ITBA), characterized by fungal invasion of respiratory tissues and potential dissemination to other organs [2]. These invasive forms predominantly affect immunocompromised individuals, including those undergoing chemotherapy, organ transplant recipients, and patients with prolonged neutropenia or on high-dose corticosteroids. ITBA, a relatively rare subtype of invasive aspergillosis, is distinguished by the specific invasion of Aspergillus species into the tracheal and bronchial tissues [2]. This condition can be particularly challenging to diagnose due to its nonspecific clinical presentation and the limitations of current diagnostic modalities.

In this report, we present a fatal case of ITBA in a patient with a history of mediastinal lung adenocarcinoma. This case underscores the importance of maintaining a high index of suspicion for invasive fungal infections in at-risk populations, particularly those with underlying malignancies or who have undergone intensive cancer therapies.

## Case presentation

A 75-year-old male with a complex medical history of mediastinal lung adenocarcinoma, radiation pneumonitis, and pulmonary nocardiosis presented to our tertiary care facility with a two-week history of progressive dyspnea. The patient's significant medical history included type 2 diabetes mellitus, chronic obstructive pulmonary disease (COPD), and a 15-pack-year smoking history.

**Figure 1:**
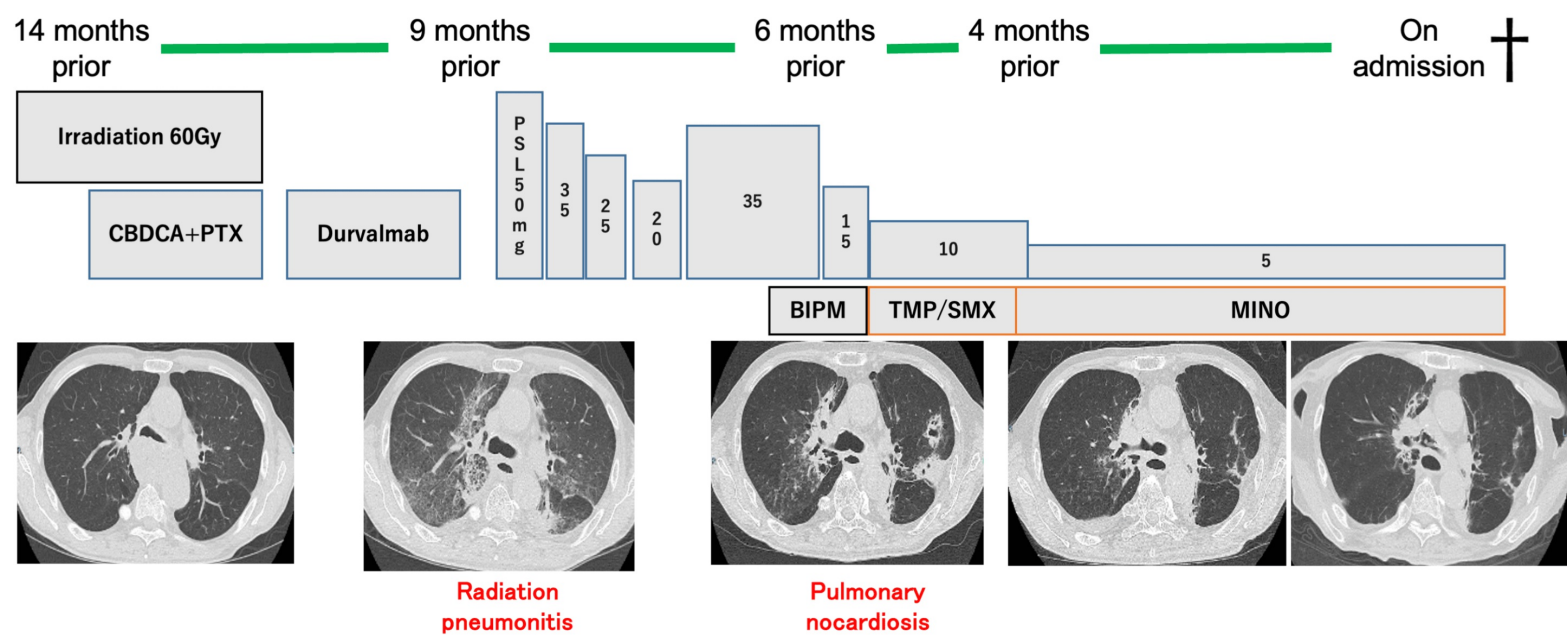
Clinical course of the patient The timeline shows the patient's treatment regimen and major clinical events from 14 months prior to admission until death. Treatment modalities are indicated in boxes: CBDCA+PTX (carboplatin plus paclitaxel), durvalumab, irradiation (60Gy), and corticosteroid doses (in mg). Antibiotics for nocardiosis are shown in orange: BIPM (biapenem), TMP/SMX (trimethoprim/sulfamethoxazole), and MINO (minocycline).

Fourteen months prior to admission, the patient was diagnosed with mediastinal lung adenocarcinoma (cT4N1M0, stage IIIA) (Figure 1). He underwent chemoradiotherapy (carboplatin plus paclitaxel with 60 Gy of irradiation), followed by four courses of durvalumab. During treatment, the patient developed COVID-19. Despite achieving a good response to chemoradiotherapy, the patient developed grade 3 radiation pneumonitis nine months before the current admission. This complication necessitated the initiation of high-dose corticosteroid therapy (prednisolone 50 mg/day), which was gradually tapered to a maintenance dose of 5 mg/day. Six months prior to the current admission, the patient developed progressive infiltrative consolidations, cavitary lesions, and nodular opacities. A transbronchial biopsy confirmed a diagnosis of pulmonary nocardiosis. Treatment with intravenous biapenem (0.6 g/day) was initiated, followed by oral minocycline (200 mg/day), which stabilized the infection.

**Figure 2:**
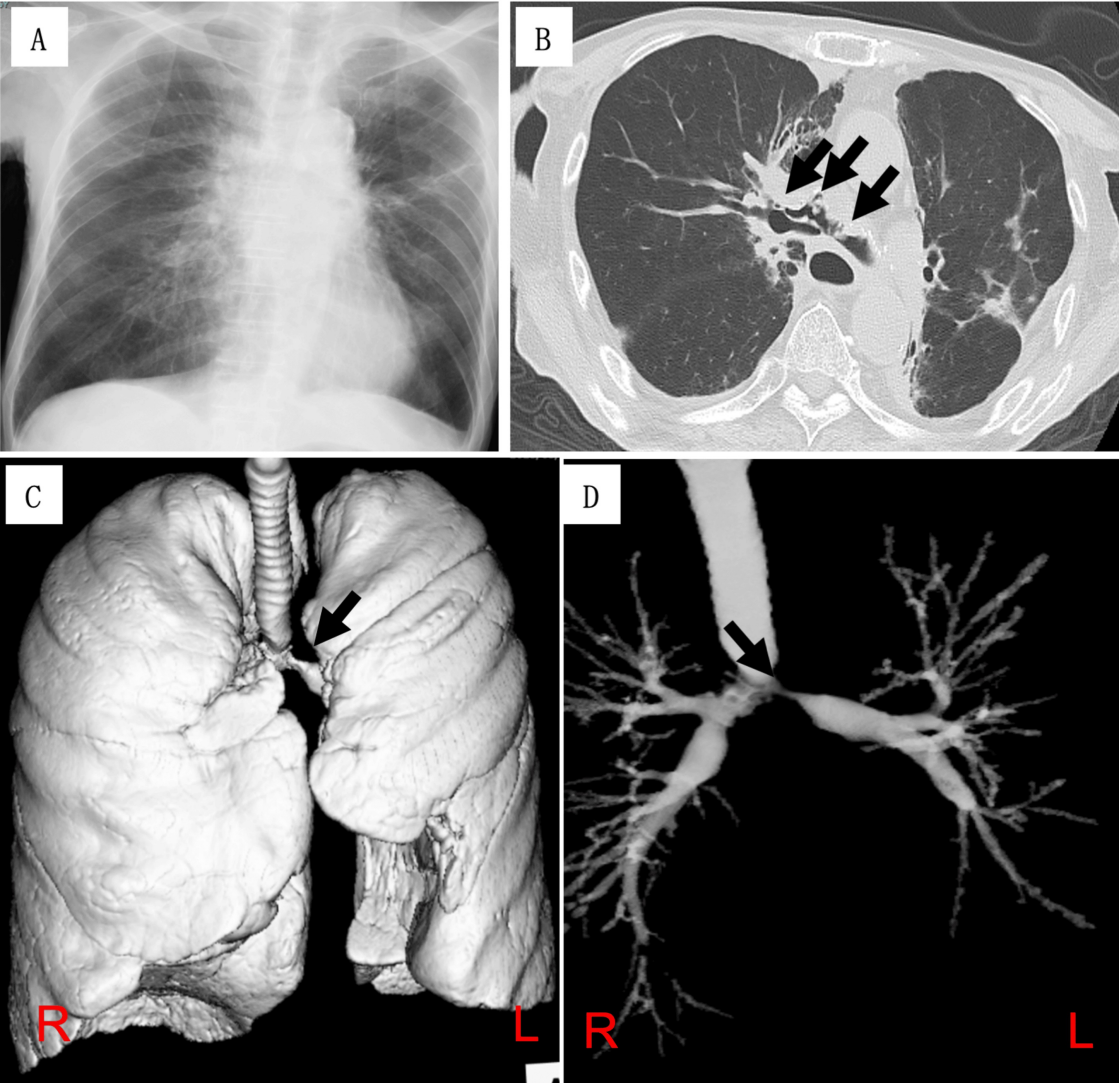
Imaging findings at admission (A) Chest X-ray showing a linear shadow and ground-glass opacity in the left upper lung field, with no significant changes compared to three months prior; (B) Chest CT revealing marked stenosis of the tracheal bifurcation and bilateral main bronchi, with extratracheal air (arrows); (C) 3D imaging (VR) demonstrating marked stenosis of the left main bronchus (arrow); (D) Isolated tracheal imaging showing more pronounced stenosis of the left main bronchus (arrow). R and L indicate the right and left sides, respectively.

On admission, vital signs were as follows: temperature 36.7°C, blood pressure 127/88 mmHg, heart rate 130 beats/min, oxygen saturation (SpO2) 99% (on 6 L/min oxygen via mask), and respiratory rate 24 breaths/min. The patient's BMI was 16.4 kg/m2 (height 159 cm, weight 41.6 kg). Chest auscultation revealed prominent stenotic sounds. Laboratory studies showed an elevated C-reactive protein (13.7 mg/dL) but normal β-D-glucan levels (12.3 pg/mL) (Table 1). Chest radiography demonstrated linear and ground-glass opacities in the left upper lung field, consistent with previous imaging (Figure 2A). Chest computed tomography (CT) revealed severe stenosis from the tracheal bifurcation to the bilateral main bronchial inlets, with evidence of extratracheal air (Figure 2B). Three-dimensional reconstruction and isolated tracheal imaging highlighted marked stenosis of the left main bronchus (Figures 2C, 2D).

**Table 1:** Serological examination of the patient on admission Plt: platelets; TP: total protein; Alb: albumin; AST: aspartate aminotransferase; ALT: alanine aminotransferase; LDH: lactate dehydrogenase; Cre: creatinine

Test	Value	Units	Test	Value	Units
WBC	6600	/μL	HbA1c	6.1	%
RBC	462	10^4/μL	CRP	13.7	mg/dL
Hb	13.7	g/dL	CYFRA	7.2	ng/mL
Plt	560	10^3/μL	D-dimer	1.8	µg/mL
TP	5.7	g/dL	β-D-glucan	12.3	pg/mL
Alb	2.4	mg/dL	NT-proBNP	727	pg/mL
AST	49	U/L	pH	7.185	–
ALT	39	U/L	PaO2	110	Torr
LDH	272	U/L	PaCO2	24.4	Torr
Cre	1.11	Mg/dL	HCO3-	8.9	mmol/L

**Figure 3:**
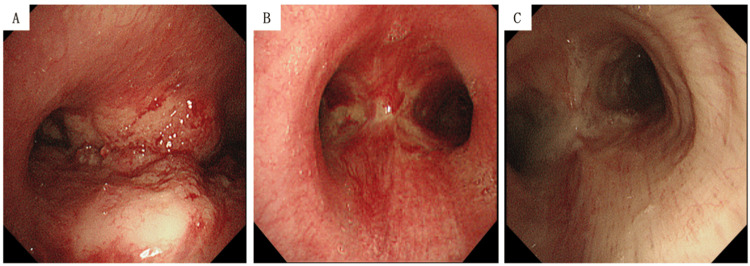
Bronchoscopic findings at different time points (A) At initial diagnosis: marginal irregular tumor causing stenosis at the tracheal bifurcation; (B) During radiation pneumonitis: shrinkage of the tumor at the tracheal bifurcation, with noticeable redness of the mucous membrane; (C) During Nocardia pneumonia: No visible tumor or mucosal redness at the tracheal bifurcation, but evidence of scarring

Based on the CT findings and previous bronchoscopic imaging (Figures 3A-3C), the differential diagnosis included recurrent lung adenocarcinoma and airway obstruction secondary to post-radiation tissue fragility. Given the patient’s compromised respiratory status and the high risk associated with bronchoscopy, a palliative approach with continuous intravenous morphine was initiated after a thorough discussion with the patient and family. The patient succumbed to his illness on the third day of admission.

**Figure 4:**
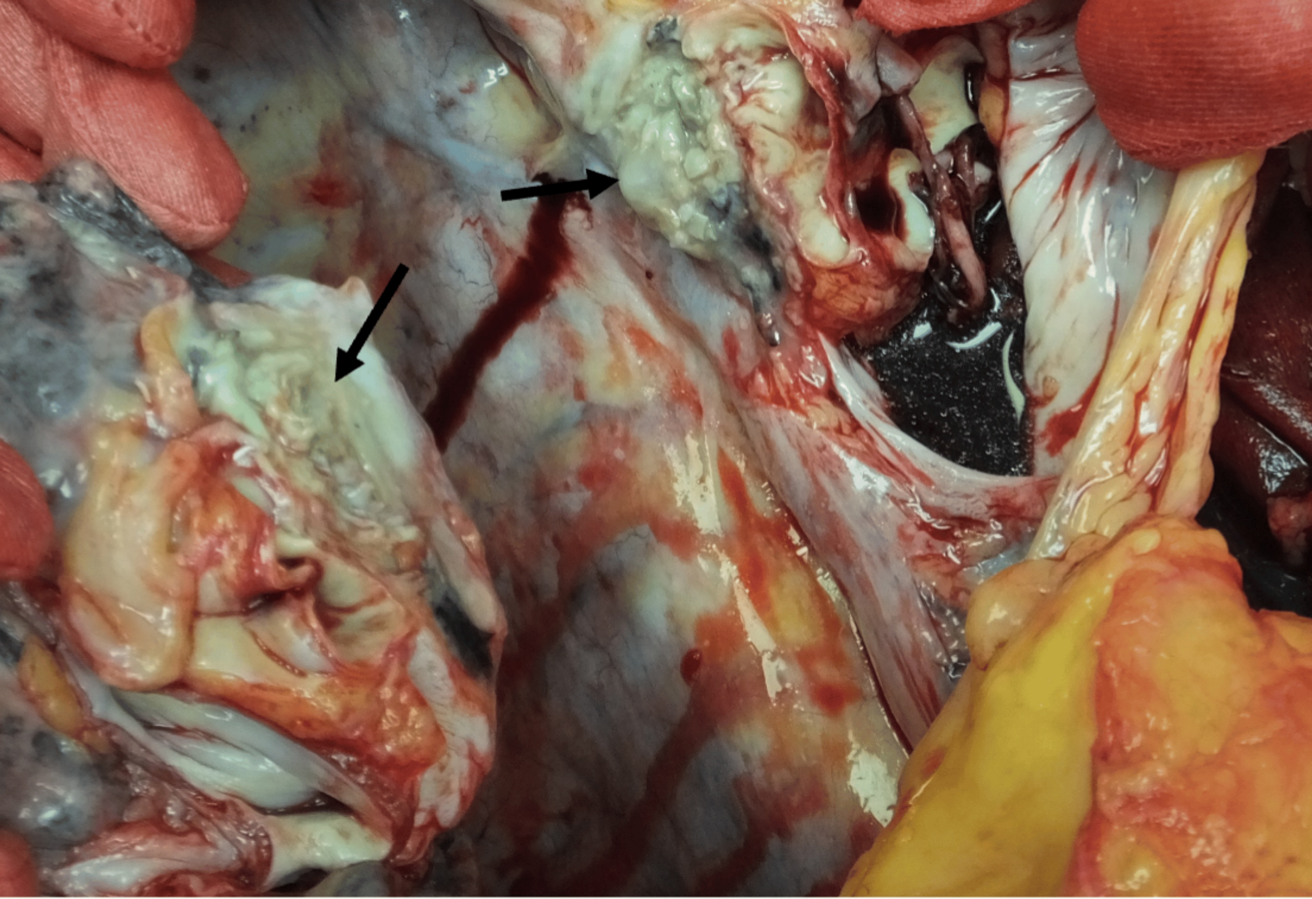
Thoracotomy findings Gross examination revealing a narrowing of the right main bronchial lumen by a grayish-white soft mass (arrows). This mass was later confirmed to be invasive tracheobronchial aspergillosis upon histopathological examination.

**Figure 5:**
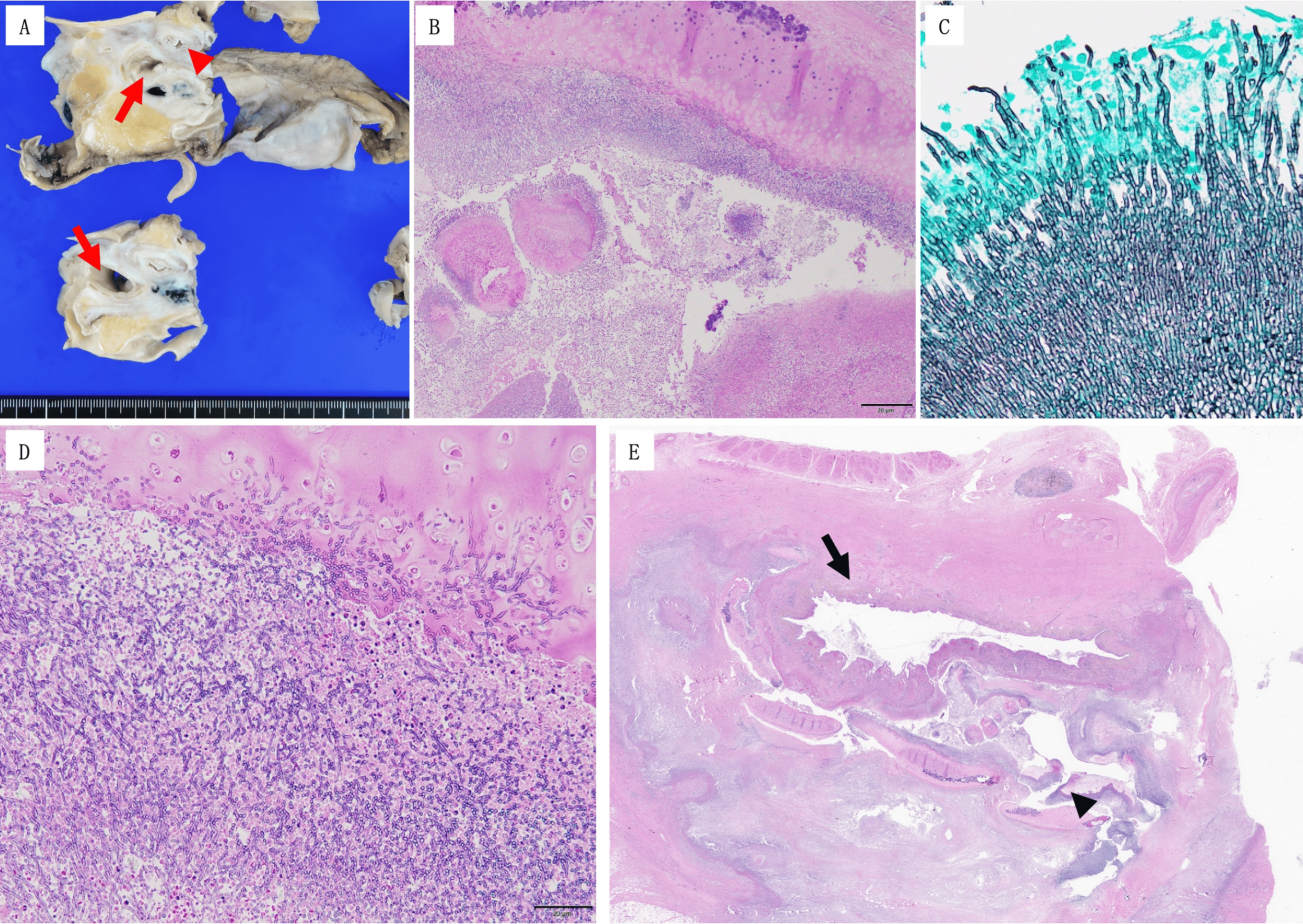
Pathological findings of invasive tracheobronchial aspergillosis (A) Gross pathology showing the tracheal bifurcation. The right main bronchus is visible (arrows) while the left main bronchus is obscured by a gray-white mass (arrowheads); (B) H&E staining of the left main bronchus showing a fungal mass in the lumen and indistinct epithelium; (C) Grocott staining revealing fungal hyphae with characteristic sharp-angle branching and septation, confirming Aspergillus infection; (D) H&E staining demonstrating Aspergillus infiltration into the bronchial cartilage; (E) Low-power view of the tracheal bifurcation showing Aspergillus growth, fungal masses, and necrosis. The left main bronchus is destroyed, obstructed, and perforated (arrowhead). The right main bronchus is narrowed at the dissection site (arrow). No residual tumor was identified.

A post-mortem examination was performed with his family’s consent. Thoracotomy revealed a pale-yellow pleural effusion and a soft, grayish-white tumor narrowing the right main bronchial lumen (Figure 4). Gross examination showed obstructive material at the tracheal bifurcation extending into both main bronchi (Figure 5A). Microscopic analysis of the left main bronchus demonstrated loss of epithelial integrity and intraluminal fungal masses (Figure 5B). Grocott staining revealed septate hyphae with acute-angle branching (Figure 5C), consistent with Aspergillus infection, which was later confirmed as Aspergillus penicillioides by ribosomal RNA sequencing. Evidence of cartilage invasion by Aspergillus was noted (Figure 5D). Extensive Aspergillus growth, fungal masses, and necrosis were observed at the tracheal bifurcation, with destruction, obstruction, and perforation of the left main bronchus and stenosis of the right main bronchus at the site of detachment (Figure 5E).

## Discussion

The advent of widespread bronchoscopy has significantly increased the recognition of ITBA in recent years [2]. Although relatively uncommon, accounting for approximately 10% of invasive aspergillosis cases [4,5], ITBA represents a critical clinical entity characterized by fungal tissue invasion. It is important to distinguish ITBA from endobronchial aspergilloma, a rare form of pulmonary aspergillosis that develops within the bronchial lumen without tissue invasion [3]. In the present case, autopsy findings conclusively demonstrated ITBA as the primary condition, evidenced by destruction, obstruction, and perforation of the left main bronchus, as well as obstruction of the right main bronchus by Aspergillus with clear tissue invasion.

ITBA is classified into three distinct subtypes: pseudomembranous, obstructive, and ulcerative tracheobronchitis [2,6]. These are characterized by bronchial inflammation due to pseudomembranes composed of Aspergillus hyphae and necrotic tissue, airway obstruction by Aspergillus mucous plugs, and invasion of the tracheobronchial mucosa or cartilage, respectively. The case presented here exhibited features of both ulcerative and obstructive tracheobronchitis in the central airways at the tracheal bifurcation, likely contributing to the rapid deterioration of the patient’s respiratory function. It is noteworthy that while ITBA can present with fever, cough, wheezing, and hemoptysis, approximately 20% of cases remain asymptomatic [7].

Kemper et al. reported that approximately 73% of ITBA patients are immunocompromised [8]. However, ITBA can also occur in patients with diabetes, lung cancer, or alcoholism, and even in immunocompetent individuals. Fernández-Ruiz et al. identified common underlying conditions including post-organ transplantation (44.2%), hematologic malignancies (21.2%), and COPD (15.4%), with a high prevalence of long-term steroid therapy (71.8%) and chemotherapy (25.0%) [9]. In the case presented, multiple risk factors likely contributed to the development of ITBA, including diabetes, COPD, prolonged steroid use, prior COVID-19 infection, nocardiosis, the original site of lung cancer, and history of radiation therapy.

Recent literature has reported cases of ITBA associated with COVID-19 infection [10], and invasive pulmonary aspergillosis has been diagnosed at autopsy in patients with severe post-COVID-19 pneumonia [11]. Notably, Aspergillus penicillioides, the strain detected in this case by ribosomal RNA sequencing, has been previously identified in such cases through nucleotide sequencing and Basic Local Alignment Search Tool (BLAST) analysis.

A review of 59 cases of pulmonary nocardiosis revealed that all patients with cavitary lesions had coinfection with Aspergillus or Mycobacterium, with multivariate analysis demonstrating a strong association between pulmonary aspergillosis and mortality risk [12]. In the present case, cavitation was observed at the onset of pulmonary nocardiosis, with initial detection of Aspergillus in sputum and slightly elevated Aspergillus antigen levels. However, subsequent negative Aspergillus antigen tests and improvement in pulmonary shadows led to continued monitoring without specific antifungal intervention.

The development of allergic aspergillosis following radiation therapy for lung cancer has been previously reported, with Aspergillus saprophytically colonizing and proliferating in locally damaged bronchial tissue corresponding to the irradiated area [13]. In this case, the patient’s history of radiation therapy (60Gy/25Fr) may have contributed to the development of ITBA. Given these multiple risk factors, it is likely that Aspergillus activity increased primarily around the tracheal bifurcation, with the accumulation of risk factors leading to the development and progression of ITBA centered at this site. The first-line treatment for ITBA is voriconazole, in accordance with guidelines for invasive pulmonary aspergillosis. Prognosis is generally poor when lesions extend deep into the bronchial wall [14].

## Conclusions

This case report highlights the importance of considering ITBA in patients with complex medical histories and multiple risk factors. The rapid progression and fatal outcome demonstrate the challenges in diagnosing and managing this rare condition. ITBA should be included in the differential diagnosis for patients presenting with airway obstruction, especially those with a history of immunosuppression, malignancy, or radiation therapy.
